# Psychometric properties of patient-reported outcome measures for dysphagia in head and neck cancer: a systematic review protocol using COSMIN methodology

**DOI:** 10.1186/s13643-022-01903-w

**Published:** 2022-02-15

**Authors:** Beatrice Manduchi, Zhiyao Che, Margaret I. Fitch, Jolie Ringash, Doris Howell, Rosemary Martino

**Affiliations:** 1 Rehabilitation Sciences Institute, University of Toronto, Toronto, ON Canada; 2 Department of Speech-Language Pathology, University of Toronto, Toronto, ON Canada; 3 The Swallowing Lab, University of Toronto, Toronto, ON Canada; 4 Bloomberg Faculty of Nursing, University of Toronto, Toronto, ON Canada; 5 Department of Radiation Oncology, Princess Margaret Cancer Centre, University of Toronto, Toronto, ON Canada; 6 Department of Otolaryngology- Head and Neck Surgery, University of Toronto, Toronto, ON Canada; 7 Princess Margaret Cancer Centre, Toronto, ON Canada; 8 University of Toronto, Toronto, ON Canada; 9 Krembil Research Institute, University Health Network, Toronto, ON Canada

**Keywords:** Deglutition disorders, Dysphagia, Swallowing, Head and neck cancer, Patient-reported outcomes, Questionnaires, Psychometric properties, Quality of life, Functional status

## Abstract

**Background:**

Dysphagia (swallowing difficulty) is one of the most common and debilitating sequelae of head and neck cancer (HNC). Patient-reported outcome measures (PROMs) are a fundamental component of dysphagia outcomes evaluation, as they inform treatment consequences that cannot be captured by objective clinician measures. Many PROMs for dysphagia in HNC are available, but their validity is unclear. As a consequence, the selection of the most appropriate PROM for dysphagia in HNC is complex and often based on the clinician’s personal preferences, rather than on valid psychometric properties. This protocol describes a systematic review aiming at (1) identifying PROMs specific to dysphagia symptoms, swallowing functional status, swallowing-related health status, and swallowing-related quality of life in HNC, (2) mapping them to our conceptual framework of dysphagia-related PROs, and (3) appraising their psychometric properties using the Consensus Based Standards for the Selection of Health Measurement Instrument (COSMIN) methodology.

**Methods:**

Six electronic databases will be searched from inception to December 2020 for all primary studies in any language and design detailing PROM development, reliability, validity, feasibility, interpretability, and/or cross-cultural adaptation. Eligibility criteria will target PROMs for patients with HNC (≥ 90% of the study sample) with ≥ 20% of their items pertaining to swallowing. Two independent raters will screen abstract and full texts and a third rater will resolve discrepancies. Data will be extracted on study, sample and PROM characteristics, and results of psychometric testing. PROMs will be mapped to our conceptual framework. The methodological quality of included PROMs and their psychometric properties will be appraised using the COSMIN risk of bias checklist and evidence will be summarized using a modified Grading of Recommendations Assessment, Development and Evaluation (GRADE) approach.

**Discussion:**

This systematic review will provide a summary of existing dysphagia-related PROMs for people with HNC and a comprehensive account of their psychometric properties. We will provide recommendations on PROMs selection which will aid healthcare professionals to the most appropriate PROM based on its validity, reliability, feasibility, interpretability and suitability for clinical and research settings. Further recommendations will be made on areas of measurement property requiring further testing.

**Systematic review registration:**

PROSPERO registration ID: CRD42021237877

**Supplementary Information:**

The online version contains supplementary material available at 10.1186/s13643-022-01903-w.

## Background

### Introduction

Head and neck cancers (HNC) are a group of tumors arising in the oral cavity, oropharynx, hypopharynx, larynx, nasopharynx, nasal cavity, and paranasal sinuses [1] and represent the sixth most frequent malignancy worldwide [2]. Organ-preserving treatments, including radiotherapy +/− chemotherapy (RT +/− CT) and minimally invasive surgical technique, are the preferred treatment approaches for early-stage HNC, while combined treatments are typically employed for advanced-stage tumors. Due to the anatomical location, treatment modalities for HNCs may significantly impact critical functions of the upper aerodigestive tract, such as eating, swallowing, speaking, and breathing.

Dysphagia (swallowing difficulty) is one of the most common and burdensome sequalae of HNC and can arise before, during, or after cancer treatment [3]. Dysphagia may lead to several medical complications, including feeding-tube dependence, pneumonia, and death [4]. Dysphagia also has detrimental emotional and psychosocial effects, leading to reduced quality of life (QoL) and poor overall wellbeing [5, 6]. Being able to accurately measure dysphagia-related outcomes is critical for the assessment of the effectiveness of dysphagia treatments in HNC, to predict QoL outcomes and to facilitate the development of dysphagia interventions based on patients’ needs and preferences [7].

### Conceptual framework of patient-reported dysphagia-related outcomes

**Fig. 1:**
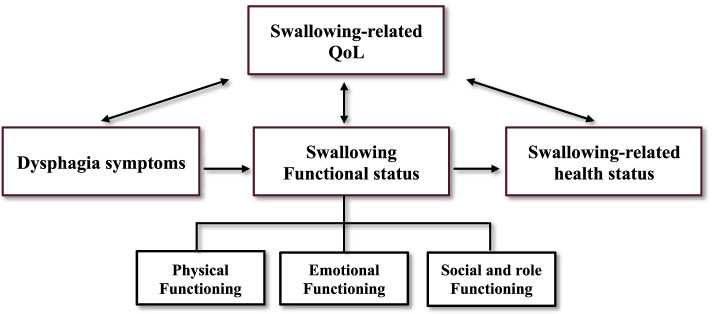
Conceptual framework of dysphagia-related patient-reported outcomes

Medical outcomes are broad in scope and encompass any marker of clinical status [8]. Given that dysphagia-related outcomes are those based on clinician and/or patient-report, they fall under the umbrella of clinical outcomes [9]. Specific to dysphagia, a conceptual framework was developed, where clinical outcomes include variables related to physiological, symptoms, functional status, health status, and QoL [10, 11]. This framework stems from the classification of health-related quality of life by Wilson and Cleary [12]. According to this framework, biological and physiological variables represent any function at the level of cells, organs, or organ system that may impact the swallowing mechanisms (e.g., HNC, or tissue fibrosis) and derive from clinician reports. Since the current study is focused specifically on patient-reported dysphagia-related outcomes, our working framework comprise those outcomes that are best determined by the person who has dysphagia and, thus, can be regarded as dysphagia-related patient-reported outcomes (PROs), i.e., dysphagia symptoms, swallowing functional status, swallowing-related health status and swallowing-related QoL (Fig. 1).

In our working framework of dysphagia-related PROs, dysphagia symptoms are defined as the patient’s perception of an abnormal state of his/her swallowing, (e.g., weight loss, or difficulty in swallowing liquids). Dysphagia symptoms may determine swallowing functional status. This is defined as the impact of dysphagia on diverse functional aspects or tasks, including physical functioning (e.g., prolonged mealtimes, diet restrictions), emotional functioning (e.g., decreased self-esteem), and social and role functioning (e.g., avoidance of eating in public). Dysphagia functional status may determine a subsequent dysphagia-related outcome, namely the patient’s perception of his/her own swallowing-related health status. Swallowing-related QoL is defined as the consequences of dysphagia on a person’s well-being. This construct holds a bidirectional relationship with the other variables. For example, functional status may determine changes in swallowing-related QoL, and changes in swallowing-related QoL can in turn affect the individual’s functional status.

Patient-reported outcome measures (PROMs) are the tools employed to measure PROs and represent a fundamental component of dysphagia outcomes evaluation, as they inform treatment harms and benefits that cannot be captured by objective measures [13]. Studies investigating the association between objective measures and PROMs in different cancer populations [14, 15] and HNC [16, 17] generally show a poor association between the two type of measures and a tendency for clinicians to underestimate symptoms severity, highlighting the importance of PROMs collection in both research and clinical settings.

### Current panorama of dysphagia-related PROMs in HNC

Currently, several tools are implemented in HNC clinical practice and research to evaluate patient-reported outcomes [18]. PROMs in HNC can be generally classified in three main groups: (i) generic PROMs, which assess patient-reported outcomes in a broad spectrum of pathologies, e.g., generic health status questionnaires such as the SF-36 [19]; (ii) disease-specific PROMs, developed specifically for HNC and intended to assess all sequalae related to HNC, e.g., pain, fatigue, sleep disturbances, dysphagia, etc., such as the FACT-H&N [20]; and (iii) symptom-specific PROMs, targeting one specific HNC-related sequalae, e.g., neck pain, such as the Neck Disability Index [21], or dysphagia, such as the M.D. Anderson Dysphagia Inventory (MDADI) [22].

Many of these instruments for HNC have been developed for, or include the assessment of, dysphagia. However, these tools target different constructs (e.g., QoL, symptoms, health status or functional status), and often the construct assessed is not explicit. As a consequence, selection of the most appropriate tool for a given clinical or research purpose is arduous, and multiple instruments are often required to address the multifactorial nature of dysphagia outcomes in HNC [23].

### Psychometric properties of dysphagia-related PROMs in HNC

The psychometric properties of a tool determine the degree to which it reflects the construct it aims to measure (content, construct and/or criterion validity), its ability to be stable over time (reliability), and its ability to detect changes over time (responsiveness) [24]. Therefore, a PROM can be considered relevant, valid and reliable only if it has proven psychometric qualities for all these dimensions.

Systematic reviews have been conducted to assess the psychometric properties of PROMs in dysphagia. However, some of these reviews included tools for dysphagia used across different populations, not specific HNC [25–27]. Other reviews focused on tools specific to HNC, but not to dysphagia [28–31]. Others established an exhaustive inventory to classify all tools commonly used in HNC according to the International Classification of Functioning, Disability and Health (ICF) but provided no qualitative appraisal [23]. As a consequence, to date, there is no critical appraisal of the psychometric properties for the available PROMs specific to dysphagia and HNC.

### Rationale and aim of the study

Despite the importance of dysphagia PROMs, approaches to dysphagia measurement in HNC are imperfect. The selection of the most appropriate PROM is complex, time-consuming, and often based on the clinician’s personal preferences, rather than on valid psychometric properties [23, 32]. It is important to not only identify all dysphagia-related PROMs currently available for HNC but to also carefully evaluate whether these tools are psychometrically sound. To address this gap, the aim of this systematic review is to (1) identify PROMs specific to dysphagia-related symptoms, swallowing functional status, swallowing-related health status, and QoL in people with HNC; (2) map each identified PROM to our working framework (Fig. 1); and, (3) critically appraise the methodological and psychometric property of each.

## Methods/design

This protocol has been developed according to the Preferred Reporting Items for Systematic reviews and Meta-Analyses Protocols guidelines (PRISMA-P; see “Additional file 1”). In addition, this review will comply with the Cochrane Methodology for systematic review [33].

The COnsensus-based Standards for the selection of health Measurement INstruments (COSMIN) Group developed a guideline for conducting systematic reviews on PROMs and provided tools to appraise the quality of their measurement properties [34]. These guidelines will be followed throughout this systematic review. The COSMIN taxonomy identifies three quality domains (i.e., reliability, validity, responsiveness) which must be appraised when assessing the quality of a PROM [35]. “Reliability” refers to the degree to which the measurement is free from measurement error, and comprises internal consistency, reliability (test-retest), and measurement error (test-retest). Other types of reliability (i.e., intra-rater and inter-rater) are not included in the taxonomy since not relevant to patient-reported instruments [34]. “Validity” refers to the degree to which an instrument measures the construct(s) it is supposed to measure. This domain encompasses construct validity (comprised of structural, concurrent, discriminant and cross-cultural validity) and criterion validity [35]. “Responsiveness” refers to the ability of an instrument to detect change over time in groups known to have experienced change [35]. Though not considered psychometric properties, two additional characteristics, namely interpretability and feasibility, are important to consider when describing a measurement tool [34]. The former refers to the degree to which a PROM’s score (or change in score) can be assigned a qualitative meaning (e.g., minimal important change, floor and ceiling effect, distribution of scores, etc.). The latter refers to the ease of application of the PROM and includes characteristics such as completion time, type and ease of administration, and ease of score calculation [34].

### Eligibility criteria

Articles will be included in this review if the focus of the research is (a) psychometric testing of an eligible PROM related to dysphagia and if ≥ 90% of their sample comprises adults (≥ 18 years old) with HNC or (b) the development of an eligible PROM related to dysphagia for HNC. We operationally define HNC to include any of the following: oral cavity (lips, floor of mouth, oral tongue, buccal mucosa, gingival, retromolar trigon, hard palate); oropharynx (tonsil, soft palate, base of tongue, posterior pharyngeal wall); nasopharynx; hypopharynx (including pyriform sinus, postcricoid region, and the pharyngeal wall); larynx (including the glottic, supraglottic, and subglottic spaces); nasal cavity and paranasal sinuses; salivary glands (parotid, submandibular, sublingual, minor salivary glands); and unknown primary cancer of the head and neck [1]. We operationally define dysphagia as swallowing disorders pertaining to the upper aerodigestive tract (from the anterior lips to the upper esophageal sphincter); this encompasses issues related to mastication and chewing and excludes issues related to dentures fit, mucositis, xerostomia, dysgeusia, pain in the mouth/ throat, esophageal dysphagia, and laryngopharyngeal reflux disease. Eligible PROMs must be related to both dysphagia and HNC and must address any of the following constructs: dysphagia symptoms, swallowing functional status, swallowing-related health stats and/or swallowing-related QoL. Psychometric testing of a PROM may include any of the following: content validity (i.e., development, content, face validity), internal structure (i.e., structural validity, internal consistency, cross-cultural validity/ measurement invariance), reliability, measurement error, validity (i.e., criterion, construct, responsiveness), interpretability (e.g., evaluating the distribution of scores, percentage of missing items, floor and ceiling effects, minimal important difference), and/or feasibility.

### Search strategy

The following databases will be searched: MEDLINE, EMBASE, CINAHL, HaNDLE-on-QoL, Cochrane Library, and PsycINFO. The search terms for “head and neck cancer AND psychometric properties AND patient-reported outcome measures” will be entered into the controlled vocabulary used for indexing specific to each database, and the terms will be combined using standard Boolean operators “OR” and “AND.” All databases will be searched from inception to December 2020 with no language restrictions. The HaNDLE-on-QoL (http://www.handle-on-qol.com/Index.aspx) is an online registry indexing papers published from 1982 to present focused on QoL in head and neck cancer that have used questionnaires. All entries indexed under the theme “Questionnaire development/Questionnaire validation” will be screened against eligibility criteria [36]. An example of database-specific (MEDLINE) search strategy is given in Appendix.

To ensure comprehensive capture of the existing literature and thorough review of tools, we will search for additional eligible articles in reference lists of all included articles and reach out to authors to obtain copies of PROM not otherwise available.

### Studies selection and data extraction

The study selection process will be systematic as per PRISMA guidelines and detailed in a flow diagram [37]. Specifically, search results will be exported into Endnote X9 and duplicates will be deleted. Full citations including their abstracts will then be exported into the Covidence platform.

Using the Covidence software, two independent reviewers will apply exclusion criteria to screen titles and abstracts to determine eligibility. Intra-rater reliability will be assessed every 200 citations to ensure consistent application of exclusion criteria. Citations will be excluded if they have no abstract or are any of the following: systematic reviews or meta-analyses; reports, editorials, textbook chapters or letters; conference proceedings; and case series with < 10 participants. Any other study type will be included in this review (i.e., randomized controlled trials, quasi-randomized controlled trials, prospective and retrospective observational studies). Studies will also be excluded if: they include no living human subjects; subjects are < 18 years old; > 10% of the sample are patients without HNC; they do not specifically target any of the following: PROM content validity, internal structure, reliability, measurement error, validity, interpretability, and/or feasibility.

All remaining citations will move to full article review. One rater will screen all articles for eligibility. A second rater will independently review 20% of the full articles to check for inter-rater reliability using Cohen’s *κ* statistic. If *κ* estimates are ≥ 0.80, we will assume that full article review was sufficient. On the other hand, if the *κ* estimates are < 0.80, the definitions and application of exclusion criteria will be reviewed and subsequently applied to an additional 20% of the full articles. Full articles will be excluded if they meet any of the exclusion criteria for citations, or for any of the following reasons: they do not include raw data related to PROM content validity, internal structure, reliability, measurement error, validity, interpretability and/or feasibility; the PROM does not contain a minimum of 20% items related to the construct of interest (i.e., dysphagia symptoms, swallowing functional status, swallowing-related health status or swallowing-related QoL); and/or the included PROM has been replaced with a newer version within the past 5 or more years. The 20% item cut-off was consensus-based with our research group. The percentage of dysphagia-related items (i.e., construct of interest) in each PROM was calculated based on the formula: $\frac{\mathrm{dysphagia}\ \mathrm{related}\ \mathrm{items}\times 100}{\mathrm{total}\ \mathrm{number}\ \mathrm{of}\ \mathrm{items}}$. Discrepancies at both the citation and full article review will be resolved by consensus with a third independent reviewer.

Data extraction of included studies will include details pertaining to general bibliometric details (e.g., study characteristics, sample characteristics); any reported findings related to psychometric testing of the PROM; and PROM specific characteristics such as those referring to domain investigated (e.g., QoL), stated model (i.e., reflective vs. formative), stated dimensionality (i.e., uni- vs. multi-dimensional), stated purpose (i.e., discriminative, predictive or evaluative), target population, and context of use and structure (e.g., number of items and subscales, response options, etc.).

Multiple reports of the same study will be counted as separate articles, but as a single study. Data on duplicate samples will be reported if outcomes refer to different psychometric properties testing. However, if data on the duplicate samples refer to the same psychometric property testing, the report with the highest risk of bias will be excluded.

Finally, we will map each tool identified according to our working conceptual framework of dysphagia-related patient-reported outcomes.

### Evaluation of the methodological quality of included studies

The evaluation of the measurement properties of the included PROMs will abide by the COSMIN methodology for systematic reviews on PROMs [34]. First, the COSMIN Risk of Bias Checklist will be employed to evaluate the methodological quality the of psychometric property testing applied to included studies [38]. Specifically, the checklist will be applied to assess risk of bias in the following domains: content validity (PROM development; content validity), internal structure (structural validity, internal consistency, cross-cultural validity/ measurement invariance), and remaining measurement properties (reliability, measurement errors, criterion validity, hypothesis testing for construct validity, responsiveness). Risk of bias for each domain will be rated as “very good, adequate, doubtful or inadequate.” The COSMIN group developed further guidelines to assess the content validity of PROMs [39]. According to the guidelines, content validity of a PROM must be evaluated by answering ten questions on relevance, comprehensiveness, and comprehensibility of the PROM, and rated as sufficient, insufficient, indetermined, or inconsistent. To address these points, three sequential steps will be performed: evaluation of the (i) quality of the PROM development and (ii) the quality of content validity studies on the PROM (using specific section of the COSMIN Risk of Bias Checklist); (iii) evaluation of overall content validity of the PROM based on results of previous steps and reviewers’ rating. These guidelines will be followed to evaluate the content validity of included PROMs.

Subsequently, psychometric testing results will be rated using the updated COSMIN criteria for good measurement properties [34]. According to these criteria, measurement properties will be classified as either sufficient, insufficient, or indeterminate.

Finally, all the articles relating to the same PROM will be compared for overall quality, consistency in findings, and if appropriate their data will be pooled (see the “Statistical analysis” section) and rated as sufficient, insufficient, inconsistent, or indeterminate. A modified GRADE (Grading of Recommendations Assessment, Development, and Evaluation) approach [34] will be employed to grade the overall quality of the evidence for measurement properties of each PROM with a rating of high, moderate, low, or very low. The GRADE judgments will be based on risk of bias (i.e., the methodological quality of the studies), inconsistency (i.e., unexplained inconsistency of results across studies), and imprecision (i.e., total sample size of the available studies). Since our operational definition restricts inclusion to only studies including HNC patients, the parameter “indirectness” (i.e., evidence from populations other than the population of interest) will not apply [34]. Feasibility and interpretability of included PROMs will be detailed descriptively.

Based on the findings of the highest quality for each PROM, we will generate recommendations on the most suitable PROM(s) with respect to each target construct (i.e., dysphagia symptoms, swallowing functional status, swallowing-related health status and swallowing-related QoL) for HNC patients.

### Statistical analysis

To ensure consistency across ratings for study selection, the percentage of agreement and Cohen’s κ will be calculated for abstract screening and a random 20% of the articles screened at full text level.

Results from studies describing the content validity, interpretability, and feasibility of the same PROM will be qualitatively summarized. Results from studies describing the internal structure (i.e., structural validity, internal consistency, cross-cultural validity/ measurement invariance), reliability, measurement error, and validity (i.e., criterion, construct, responsiveness) of the same PROM will be statistically pooled in meta-analysis, if the data meets our definition of homogeneous. Specifically, for each psychometric property captured, data will be considered homogenous if across ≥ 2 studies they are referring to the same PROM, analyzed through the same measurement theory (i.e., CTT vs. IRT) and same statistics (e.g., Cronbach’s alpha for internal consistency testing; ICC for test-retest reliability testing) and compared against the same tool/ reference standard (this parameter will only apply to validity testing). Homogenous data will be eligible for data pooling only if they are reported as single point estimates, the sample size is specified, and at least one measures of variability is reported (including standard deviation, standard error, or confidence intervals). All eligible homogeneous data will be pooled by calculating weighted means (based on the number of participants included per study) and 95% confidence intervals. When the criteria for pooling are not met, reported results for internal structure, reliability, measurement error, and validity will qualitatively summarized studies for each PROM. Statistical analyses will be completed using RStudio software, version 1.2.5001.

## Discussion

This systematic review will provide a summary of existing dysphagia-related PROMs for people with HNC and a comprehensive assessment of available evidence specific to the psychometric properties of each one. These findings will provide an inventory of the dysphagia-related PROMs which are currently available for HNC and an account of how they map to the domains illustrated in our working framework, namely dysphagia symptoms, swallowing functional status, swallowing-related health status, and swallowing-related QoL. This inventory will shed light on the content and constructs of PROMs commonly employed in research and clinical practice and will guide the selection of the most appropriate tool fit for a given clinician’s or researcher’s purpose. Findings from this work will enable different users to have a complete understanding of psychometric properties of existing PROMs and as such will help in the selection of the most appropriate PROM based on its content, internal structure, reliability, measurement error, validity, interpretability, feasibility, and suitability for either clinical or research settings. In addition, these findings will highlight potential gaps in the quality of data about measurement properties and provide recommendations for areas requiring further testing.

Some potential challenges may arise while we are conducting this study. Although this review will follow a robust methodology and well-defined taxonomy of measurement properties, we anticipate that many studies might not have followed the COSMIN criteria for psychometric properties. This may pose a challenge during the data extraction and evaluation of methodological quality. To address this issue, we will follow the COSMIN Guidelines for systematic review on PROMs, which account for these potential challenges by providing methods to evaluate psychometric properties which were not tested according to COSMIN criteria. Furthermore, our definition of dysphagia-related outcomes might not align with the constructs assigned by the authors of included PROMs. This might pose a challenge during the study selection process. To overcome this potential issue, inclusion criteria are extended to PROMs that include any dysphagia-related question, regardless how it was originally labeled. As part of the data extraction and appraisal process in this review, we will attempt to align the PROM according to our working framework.

In conclusion, this study represents the first attempt to summarize available PROMs related to dysphagia for people with HNC and will provide a comprehensive summary of their psychometric properties. The results of this study will help users to identify the most appropriate PROMs based on their validity and suitability for clinical and research settings.

### Supplementary Information

**Additional file 1.** PRISMA-P 2015 Checklist.

## Data Availability

Not applicable

## Appendix

### Example of Database-Specific Search Strategy (MEDLINE)

(exp "Head And Neck Neoplasms"/ or HNC.kf,tw. or HNSCC.kf,tw. or SCCHN.kf,tw. or OPSCC.kf,tw. or ((cancer* or onco* or malignan* or neoplasm* or carcin* or tumo?r* or squamous cell) adj3 (head or neck or oral or mouth* or lip* or tongue* or tonsil* or palat* or pharyn* or nasopharyn* or oropharyn* or hypopharyn* or laryn* or glotti* or supraglotti* or subglotti* or nasal* or paranasal* or salivar* or parotid*)).kf,tw.) AND (validation study/ OR exp "sensitivity and specificity"/ or factor analysis, statistical/ OR "reproducibility of results"/ OR Psychometrics/ OR exp observer variation/ OR discriminant analysis/ OR Comparative Stud*.kf,tw. OR psychometric*.kf,tw. OR clinimetr*.kf,tw. OR clinometr*.kf,tw. OR reproducib*.kf,tw. OR (observer* adj3 variation*).kf,tw. OR reliab*.kf,tw. OR unreliab*.kf,tw. OR valid*.kf,tw. OR coefficient*.kf,tw. OR homogene*.kf,tw. OR (internal consistency).kf,tw. OR (cronbach* adj3 alpha*).kf,tw. OR (item* adj1 (correlation* or selection* or development* or reduction*)).kf,tw. OR agreement*.kf,tw. OR precision*.kf,tw. OR imprecision*.kf,tw. OR precise value*.kf,tw. OR (test retest*).kf,tw. OR interrater.kf,tw. OR inter-rater.kf,tw. OR intrarater.kf,tw. OR intra-rater.kf,tw. OR intertester.kf,tw. OR inter-tester.kf,tw. OR intratester.kf,tw. OR intra-tester.kf,tw. OR interobserver.kf,tw. OR inter-observer.kf,tw. OR intraobserver.kf,tw. OR intra-observer.kf,tw. OR interexaminer.kf,tw. OR inter-examiner.kf,tw. OR intraexaminer.kf,tw. OR intra-examiner.kf,tw. OR interindividual.kf,tw. OR inter-individual.kf,tw. OR intraindividual.kf,tw. OR intra-individual.kf,tw. OR kappa*.kf,tw. OR repeatab*.kf,tw. OR ((replicab* or repeated) adj1 (measure* or finding* or result* or test*)).kf,tw. OR concordance.kf,tw. OR (intraclass correlation).kf,tw. OR discriminative.kf,tw. OR (factor analys*).kf,tw. OR (factor structure*).kf,tw. OR subscal*.kf,tw. OR (interscal* correlation*).kf,tw. OR (interval variabilit*).kf,tw. OR (known group*).kf,tw. OR (individual variabilit*).kf,tw. OR (variabilit* analys*).kf,tw. OR (standard error of measurement).kf,tw. OR responsive*.kf,tw. OR (limit* detection*).kf,tw. OR interpretab*.kf,tw. OR (small* adj1 (real* or detectable*) adj1 (change* or difference*)).kf,tw. OR ((minimal* or clinical*) adj1 (important or significant* or detectable or meaningful) adj1 (change* or difference*)).kf,tw. OR (ceiling effect).kf,tw. OR (floor effect).kf,tw. OR (item response model).kf,tw. OR IRT.kf,tw. OR Rash.kf,tw. OR (differential item functioning).kf,tw. OR DIF.kf,tw. OR (cross-cultural adj1 (adapt* or equivalence)).kf,tw.) AND (Self Report/ OR Patient Reported Outcome Measures/ OR Patient Satisfaction/ OR "Surveys and Questionnaires"/ OR (PRO or PROs or PROM or PROMs).kf,tw. OR ((patient* or self) adj2 (apprais* or report* or rated or rating* or based or assess* or administ* or evaluat* or complet*)).kf,tw. or "Symptom Assessment"/ OR "Quality of Life"/ OR (HRPRO or HRQL or HRQoL or QoL).kf,tw. OR Health Status/ OR (health index* or health indices or health profile*).kf,tw. OR ((symptom* or disabilit* or function* or subjective or wellbeing or well being) adj3 (index or indices or instrument* or measure* or questionnaire* or profile* or scale* or score* or status or survey*)).kf,tw.

## Abbreviations

HNC: Head and neck cancer
RT: Radiotherapy
CT: Chemotherapy
QoL: Quality of life
PRO: Patient-reported outcome
PROM: Patient-reported outcome measure
SF-36: Short Form Health Survey – 36
FACT-H&N: Functional Assessment of Cancer Therapy - Head & Neck cancer
MDADI: M.D. Anderson Dysphagia Inventory
PRISMA-P: Preferred Reporting Items for Systematic reviews and Meta-Analyses Protocols
COSMIN: COnsensus-based Standards for the selection of health Measurement Instruments
GRADE: Grading of Recommendations Assessment, Development, and Evaluation
